# Characterization and biological applications of gonadal extract of *Paracentrotus lividus* collected along the Mediterranean coast of Alexandria, Egypt

**DOI:** 10.1371/journal.pone.0296312

**Published:** 2024-01-02

**Authors:** Nehal Shawky Nagy, Amina Essawy Essawy, Soheir Salem Al-Sherif, Mohamad Moustafa Ali, Eman Sheta Alsawy, Mohamed Helal

**Affiliations:** 1 Department of Zoology, Faculty of Science, Alexandria University, Alexandria, Egypt; 2 Department of Medical Biochemistry and Microbiology, Science for Life Laboratory, Uppsala University, Uppsala, Sweden; 3 Department of Pathology, Faculty of Medicine, Alexandria University, Alexandria, Egypt; 4 National Institute of Oceanography and Fisheries (NIOF), Cairo, Egypt; Cairo University, Faculty of Science, EGYPT

## Abstract

Marine invertebrates represent a valuable reservoir of pharmaceutical bioactive compounds with potential relevance to various medical applications. These compounds exhibit notable advantages when compared to their terrestrial counterparts, in terms of their potency, activity, and mechanism of action. Within this context, the present work aimed to extract, chemically characterize, and investigate the bioactivity of the gonadal extract of the sea urchin *Paracentrotus lividus* (*P*. *lividus*) collected along the Mediterranean coast of Alexandria, Egypt. Fractions of the gonadal extract were characterized by Spectrophotometry and gas chromatography-mass spectrometry (GC-MS), and their bioactivities were investigated in vitro. The analysis supported the extract richness of carotenoids and bioactive compounds. The extract showed promising anticancer activity against three different breast cancer cell lines with different levels of aggressiveness and causative factors, namely MDA-MB-231, MDA-MB-453, and HCC-1954. Gene expression analysis using RT-qPCR showed that *P*. *lividus* extract inhibited the expression of crucial factors involved in cell cycle regulation and apoptosis. In addition, the extract significantly inhibited the lipo-polysaccharides (LPS) induced inflammation in the RAW264.7 macrophage cell line and exerted anti-bacterial activity against the Gram-negative bacteria *Klebsiella pneumoniae* and *Pseudomonas aeruginosa*. Collectively, these results demonstrated the chemical richness and the wide-scale applicability of *P*. *lividus* gonadal extract as an anti-cancer, anti-bacterial, and anti-inflammatory natural extract.

## Introduction

The field of isolation and characterization of marine natural products is rapidly growing. It aims to isolate, detect, identify, and understand their structure and applications [1, 2]. It has been proven that these natural products have hundreds of leading bioactive compounds with high therapeutic potential. These compounds, such as carotenoids, phenolics, fatty acids, and proteins, are naturally produced from plants, animals, fungi, and microorganisms [3, 4]. These natural products possess several advantages over synthetic chemicals owing to their incomparable molecular diversity and biological functionality that enable a wide spectrum of interactions with proteins and other biological targets [5].

Sea urchins are a group of benthic echinoderms that belong to the class Echinoidea. Echinoderms are rich in bioactive metabolites, antioxidants, antimicrobial and anti-tumor compounds [6, 7]. Additionally, sea urchins are rich in minerals, proteins, vitamins, fatty acids, carotenoids, and polysaccharides that possess various biological functions [8, 9]. Of note, the hydrolysate extract of sea urchins is useful for various biomedical applications [10]. For instance, Ganglioside Hp-s1 isolated from *Hemicentrotus pulcherrimus* sperms possesses an anti-neuroinflammatory activity via NF-kB and JNK/p38 MAPK downregulation [11]. Bioactive compounds extracted from *Stomopneustes variolaris* showed antioxidant, anti-inflammatory, anti-diabetic, and anti-cancer properties [12]. Sea urchin-purified spinochromes and naphthoquinones exhibit antioxidant and cytotoxic anti-bacterial properties [13]. Interestingly, extracts from different sea urchin body parts showed different biological activities [14]. For example, extracts from the gonads, gut, spines, and mouth parts of the genus *Tripneustes gratilla* showed in vitro anti-microbial and hemolytic activities. Therefore, the diversity of bioactive molecules present within different organs of sea urchins could, in part, explain the vast reactivities among different extracts [15, 16]. Importantly, sea urchin gonads, which are half-moon-shaped and yellow to orange in color constitute only 10% of the total weight and are the edible portion. They are eaten because of their characteristic flavor, their richness of bioactive compounds (polysaccharides, fatty acids, etc.), and their medicinal properties [17, 18]. Research reports indicated that sea urchin gonads contain a high content of polyunsaturated fatty acids (PUFAs) [19], and carotenoids [20–22].

Carotenoids are the most abundant pigment groups in nature. They also form the most important compounds detected in the sea urchin gonads. The major carotenoids in sea urchin gonads are echinenone, β-carotene, canthaxanthin, and astaxanthin [22, 23]. Generally, carotenoids have valuable anti-cancer, anti-obesity, anti-diabetic, anti-inflammatory, and cardioprotective activities [24]. Carotenoids of marine origin have an anti-proliferative effect on different cancer cell lines [25]. The GC-MS analysis of the gonadal extract of the Australian purple sea urchin *Heliocidaris erythrogramma* detected different classes of fatty acids such as saturated fatty acids (SFAs), mono-unsaturated fatty acids (MUFAs,) and PUFAs. Additionally, The extract displayed significant anti-inflammatory and anti-tumor activities [26]. Moreover, the anti-oxidant, anti-diabetic, and anti-inflammatory properties of the gonad methanolic extract of the purple sea urchin *Echinometra mathaei* were experimentally evident [14]. The gonadal extract of *Diadema setosum* was effective in regulating the immune response and could be utilized as a dietary supplement for acute and chronic inflammation [27]. Similarly, PUFAs and carotenoids were detected in the gonadal extract of the green sea urchin *Strongylocentrotus droebachiensis*, where the extract demonstrated anti-inflammatory and anti-diabetic activities [28]. Among different sea urchin species, *P*. *lividus* [Lamarck, 1816] is the most appreciated one, it grows in the Atlantic and is found on the Southeastern Mediterranean coast of Alexandria, Egypt [29]. Previous reports on *P*. *lividus* extract not only demonstrated its activity against oxidative stress and liver damage in type I and type II diabetes mellitus [30] but also showed the potential use of the extract as a source of valuable low-cost collagen for mechanically resistant biomedical devices [31]. Currently, there are almost twenty marine-derived bioactive compounds available in the market [32], including Histochrome which is a sodium salt of Echinochrome-A (sea urchin pigment) and it has been in the clinic since 1999 [33]. Reports on the biological activity of different sea urchin extracts are intensively reported, however, little is known about the biological activity of the gonadal extract. Therefore, the current study aims to isolate and characterize the chemical composition of the components of the gonadal extract of *P*. *lividus* and investigates their biological properties as anti-cancer, anti-inflammatory, and antimicrobial agents.

## Material and methods

### Sample collection and extraction of bioactive compounds from gonads

Mature individuals of Paracentrotus lividus (Lamarck, 1816) were procured through underwater collection by marine divers along the coast of Abou Quir, located in Alexandria, Egypt. Following their retrieval, these sea urchins were expeditiously transported to the laboratory while still alive. To obtain the gonadal extract, sea urchins were dissected, gonads were collected, washed in seawater to remove any debris then weighed. For each 3–4 gm of weighted gonads, 10 ml of HPLC grade acetone was added and gentle homogenization was carried out. The homogenate was collected and then centrifuged for 3 min at 1500 rpm under cooling conditions. An equal volume of methyl tertiary-butyl ether (MTBE) and 5 ml of distilled water were added to it and vigorously shaken. Then the organic layer (which contains the extract) was collected, filtered, and dried under nitrogen gas evaporation. Butylhydroxytoluene, BHT (0.1%), was added to the extraction solvent to prevent carotenoid oxidation [34]. All experimental procedures were followed according to ethical principles recommended by the Alexandria University guideline for animal care with an approval code (AU 04 21 09 23 2 01).

### Gas Chromatography-Mass Spectroscopy (GC-MS) analysis of gonadal extract

The bioactive contents of the extracted gonadal extract were estimated according to gas chromatography mass-spectrometry analysis (GC2010 & GCMS-QP2010, respectively). The mass spectrometer was run at 70 eV and scanned fragments from 30 to 300 m/z. Peak identification of gonadal extract depended on comparing the gained mass spectra with NIST88 Library. Compounds were identified by comparing the retention times with those of authentic compounds and with the spectral data obtained from the data library of the corresponding compounds. Quantities of the compounds are represented as relative area percentages derived from the integrator. The injected sample volume was 1 μl.

### Carotenoid detection by spectrophotometer

The absorbance of carotenoids was examined at three distinct wavelengths, yielding spectra featuring three distinct peaks [35]. To achieve this, an acetone-based gonadal extract was meticulously prepared, and the presence of carotenoids was ascertained across a wavelength range spanning from 380 to 520 nm. This assessment was carried out utilizing a spectrophotometer (T70, PG Instruments Limited, United Kingdom)

### Anti-cancer activity of gonadal extract

Three breast cancer cell lines were used in this work (MDA-MB-231, MDA-MB-453, and HCC 1954) to investigate and study the anti-cancer properties *of P*. *lividus* gonadal extract. The MDA-MB-231 cell line was cultured in a Dulbecco’s Modified Eagle’s Medium (DMEM) (Gibco, Thermo Fisher Scientific) while MDA-MB-453 and HCC-1954 were cultured in RPMI 1640 medium (Gibco, Thermo Fisher Scientific). All cell culture media were supplemented with 10% fetal bovine serum (FBS), 2 mM L-glutamine, and 1% Pen-Strep antibiotics (Gibco, Thermo Fisher Scientific). All cells were maintained at 37°C in a humified incubator with 5% CO_2_. Different concentrations of *P*. *lividus* gonadal extract was used for the cell viability experiment to determine the 50% inhibition concentration (IC50) in each cell line. For each concentration, 2000 cells per well were seeded in a 96-well plate for 24 hours. Then, the indicated concentrations of the extract were added and incubated for 48 hours. To assess the cell viability, Presto Blue™ Cell Viability Reagent (Thermo Fisher Scientific, Cat# A13262) was used according to the manufacturer’s protocol, and the fluorescence signal at an Excitation/Emission wavelength of 560/590 nm using a microplate reader (PerkinElmer) was measured.

### Gene expression analysis

Total RNA was extracted from cells using Relia Prep™ RNA Cell Miniprep System (Promega, Cat# Z6012) according to the manufacturer’s instructions. One microgram of total RNA from each sample was subjected to cDNA synthesis utilizing a High-Capacity cDNA Reverse Transcription Kit (Applied Biosystems™, Cat# 4368814). The expression of different coding genes using quantitative real-time PCR assays performed on a Bio-Rad CFX96 cycler (Bio-Rad Laboratories Inc.) was investigated. The name and primer sequences of each tested gene and the housekeeping gene were tabulated in Table 1. For each gene, a master mix was prepared with the aid of qPCR BIO SyGreen 2× Master Mix (PCR Biosystems, Cat#PB20.14–50) following the manufacturer’s instructions. Gene fold change expression levels of different samples were calculated using the threshold cycles (CTs), also known as the 2-ΔΔ*CT* method [36].

**Table 1:** Primer pair sets of target and housekeeping genes.

Gene name	Forward primer	Reverse primer
Bcl2	AGGCTGGGATGCCTTTGTGG	TTTGTTTGGGGCAGGCATGT
ATM	CAGGGTAGTTTAGTTGAGG	CTATACTGGTGGTCAGTGC
ATR	GTTGGGCCCACTTTATGCAG	TGCTCTTTTGGTTCATGTCCAC
BRCA1	ACAGCTGTGTGGTGCTTCTGTG	CATTGTCCTCTGTCCAGGCATC
CDK1	CGCCGCGGAATAATAAGCC	AGGAACCCCTTCCTCTTCACT
CDK4	TGTTGTCCGGCTGATGGACG	CCTTGATCGTTTCGGCTGGC
CYCB1	ACCTGTGTCAGGCTTTCTCTG	TGGTCTGACTGCTTGCTCTTC
P21	CTGCCCAAGCTCTACCTTCC	CAGGTCCACATGGTCTTCCT
HPRT1(housekeeping gene)	CCCTGGCGTCGTGATTAGT	CACCCTTTCCAAATCCTCAGC

### Anti-inflammatory activity of gonadal extract

Murine macrophages RAW264.7 cells (ATCC®) were maintained in complete DMEM (Corning, USA), with 10% FBS, streptomycin sulphate (100 μg/mL), penicillin (100 U/mL), and 2 mM L-glutamine in a humidified 5% CO2 incubator. Cells were washed with phosphate-buffered saline and scrapped off the flasks using sterile scrappers (SPL, Spain). RAW 264.7 cells (0.1 × 10^6^ cells/mL) were seeded into each well of 96-well microwell plates and incubated overnight. Three groups of triplicate wells were prepared. The sample vehicle group received (DMSO, 0.1% V/v) and culture media, the inflammation group received the inflammation inducer [lipopolysaccharide (LPS) as 100 ng/mL in complete culture media], and the sample groups received concentrations (10,100, 200 μg/ml) of the extract dissolved in DMSO and diluted in culture media which contain LPS (final concentration of DMSO = 0.1% V/v). As an established positive control for the anti-inflammatory effect, Indomethacin (Indo, 0.25mM, Sigma) was used [37, 38]. After 24 h of incubation, the Griess assay [39] was performed to determine the available nitrite to indicate the breakdown of nitric oxide (NO) in all wells. Equal volumes of culture supernatants and Griess reagent were mixed and incubated at room temperature for 10 min to form the colored diazonium salt, which was detected at an absorbance of 540 nm on a Tecan Sunrise™ microplate reader (Austria). NO inhibition (%) of the tested extract was calculated relative to the inflammation group, normalized to cell viability determined with Alamar Blue™ reduction assay [40, 41].

### Anti-microbial activity of gonadal extract

The agar well diffusion assay [42, 43] was utilized to assess the antibacterial activity of the sea urchin gonadal extract. Five microbial species were screened including Gram-negative Bacteria: *Klebsiella pneumoniae ATCC700603*, *E*. *coli ATCC25922* and *Pseudomonas aeruginosa ATCC 27853*, Gram-positive Bacteria: *Streptococcus pyogenes EMCC1772* in addition to *Candida albicans EMCC105*. Briefly, the bacteria were grown in nutrient agar at 37°C for 24 hours and *Candida albicans* was grown in potato dextrose agar at 28°C for 24 hours. One hundred μl of the inoculums (1×10^8^ CFU/ml) was inculcated on the surface of the agar media. Five different concentrations of the gonadal extract were prepared in five concentrations and 100 μl were applied in a 5 mm diameter well (wells were made using a sterilized cork borer). The zone of inhibition was calculated by measuring the diameter of the inhibition zone around the well (mm), including the well diameter. The readings were taken in three different directions in all triplicates and the average values were tabulated. Amoxicillin standard 0.01% (Pharco pharmaceuticals) was used as a positive control antibiotic, while Clotrimazole standard 0.01% (Pharco pharmaceuticals) was used as a positive control antifungal agent. The minimum inhibitory concentration (MIC) was determined using the agar dilution method [44, 45], the tested extract was firstly diluted to make a series of concentrations (200 mg/ml to 25 mg/ml), an appropriate volume of each dilution was added to the melted agar to make plates (agar diluted), then the tested microorganisms were added on the surface. The lowest concentration of the extract that inhibits the visible growth of the tested microorganisms was the MIC value.

### Statistical analysis

Statistical analysis was performed using graph pad prism 7.00 software, the data were evaluated by T.test and presented as mean ± Standard deviation. Significance is marked for p-value ≤ 0.05.

## Results

### GC-MS characterization of the gonadal extract and carotenoid detection

**Fig 1:**
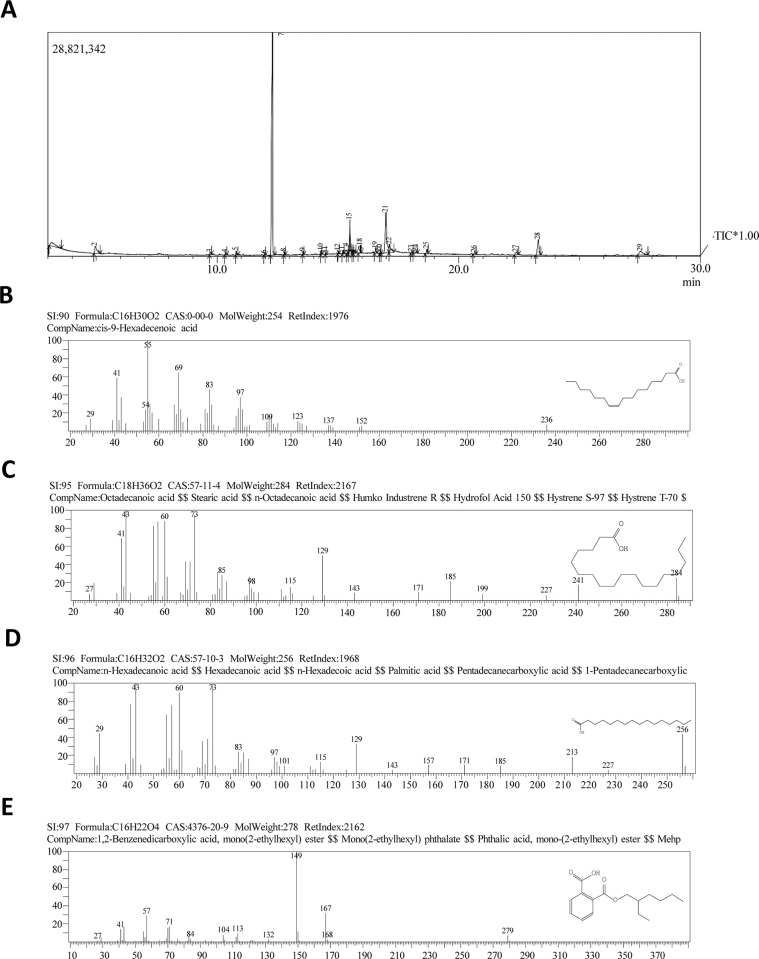
GC-MS chromatogram of *P*. *lividus* gonadal extract. Briefly: *P*. *lividus* gonads were freshly dissected under cooling conditions and extracted with acetone and Methyl tertiary-butyl ether then the organic extract was concentrated under nitrogen gas and applied to GC-MS analysis (**A**). Examples of the anti-inflammatory **B**) palmitoleic acid, anti-bacterial **C**) Octadecanoic acid, **D**) palmitic acid, and, anticancer compound **E**) 1,2-Benzenedicarboxylic acid, mono(2-ethylhexyl) ester.

As shown in Table 2, thirty compounds were identified by the Gas chromatography-mass spectrometry (GC-MS) analyses of the *P*. *lividus gonadal* extract (Fig 1 and S1 Fig). The prevalent compounds were alkane hydrocarbons (Tetradecane, Octadecane, Nonadecane), alkane (Eicosane), aldehydes (Octadecanal,), fatty acids (Octadecanoic acid “Stearic acid”, Hexadecanoic acid “palmitic acid”), fatty acid methyl esters (Octadecanoic acid-methyl ester, Hexadecanoic acid- methyl ester), 2-Undecenal, cis-9-Hexadecenoic acid (palmitoleic acid), 9-Octadecenoic acid, methyl ester, cis-Vaccenic acid, Oleyl Alcohol, cis-11-Eicosenoic acid, methyl ester, Hexadecenoic acid, 2-hydroxy-1-(hydroxymethyl) ethyl ester (2-Palmitoylglycerol), 2-(((2-Ethylhexyl)oxy)carbonyl)benzoic acid, 9-Octadecenoic acid (Z) and 2,3-hydroxypropyl ester (Glyceryl monooleate). The visible absorption data for the carotenoid compounds detected in the *P*. *lividus* gonadal extract using the spectrophotometer are shown in Table 3. All the identified compounds exhibited their maximum absorption at three specific wavelengths, whereas Astaxanthin and Echinenone displayed absorption at a single wavelength.

**Table 2:** List of detected compounds in the *P*. *lividus* gonadal extract using (GC–MS).

Constituent	R. Time	Area	MF	MW (g/mol)
**2-Undecenal**	10.789	139783	C11H20O	168
**Tetradecane**	11.965	73985	C14H30	198
**Eicosane**	13.572	134502	C20H42	282
**Octadecanal**	14.500	19683	C18H30O	268
**Octadecane**	15.018	136576	C18H36	254
**Hexadecanoic acid, methyl ester**	15.214	132442	C17H34O2	270
**Cis-9-Hexadecenoic acid (palmitoleic acid)**	15.386	45581	C16H30O2	254
**Hexadecanoic acid (palmitic acid)**	15.500	842680	C16H32O2	265
**Nonadecane**	15.707	123069	C19H40	268
**9-Octadecenoic acid, methyl ester**	16.565	122817	C19H36O2	296
**Octadecanoic acid, methyl ester**	16.748	54556	C19H38O2	298
**Cis-Vaccenic acid**	16.993	2127578	C18H34O2	282
**Octadecanoic acid (Stearic acid)**	17.142	341102	C18H36O2	284
**Oleyl Alcohol**	18.247	68322	C18H36O	268
**Cis-11-Eicosenoic acid, methyl ester**	18.692	148195	C21H40O2	324
**Hexadecanoic acid, 2-hydroxy-1-(hydroxymethyl) ethyl ester (2-Palmitoylglycerol),**	22.380	206289	C19H38O4	330
**1,2-Benzenedicarboxylic acid, mono(2-ethylhexyl) ester**	23.293	2664521	C16H22O4	278
**9-Octadecenoic acid (Z)-, 2,3-dihydroxypropyl ester (Glyceryl monooleate).**	27.526	538150	C21H40O4	356

**Table 3:** Detection of carotenoid compounds in *P*. *lividus* gonadal extract dissolved in acetone.

Carotenoid	Wavelength	Absorbance (AU)
**Lycopene**	448	1.146
	474	1.456
	505	1.494
**Neoxanthin**	416	0.923
	440	1.088
	470	1.395
**Zeaxanthin**	430	1.019
	452	1.153
	479	1.466
**Astaxanthin**	480	1.460
**Auroxanthin**	381	0.585
	402	0.696
	427	0.987
**α-carotene**	424	0.964
	448	1.105
	476	1.300
**β-carotene**	429	0.972
	452	1.103
	478	1.397
**γ-carotene**	439	1.001
	461	1.171
	491	1.356
**echinenone**	460	1.140

### Gonadal extract of *P*. *lividus* affects the viability and gene expression of breast cancer cells

**Fig 2:**
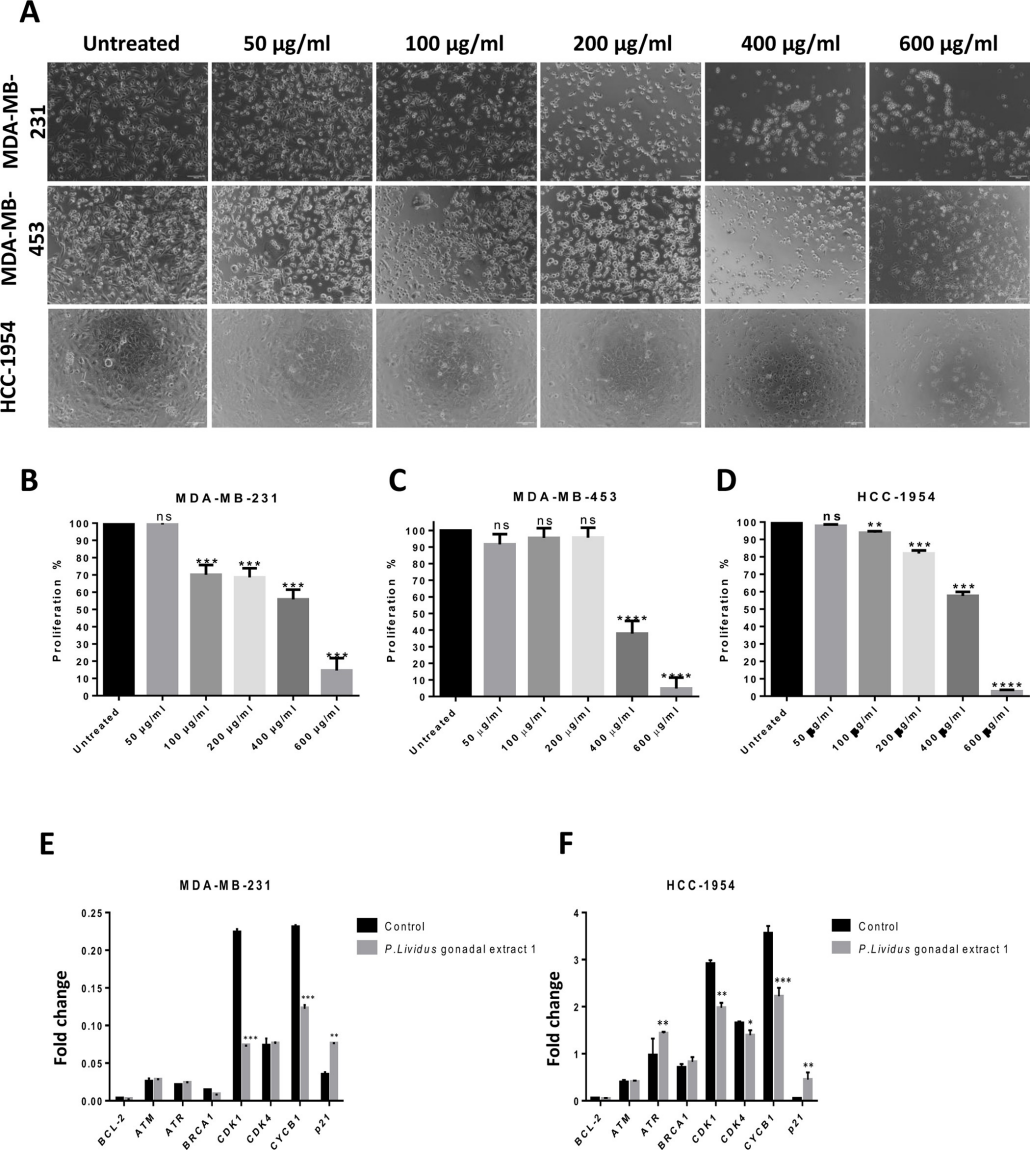
Invitro anticancer activity of *P*. *lividus* gonad extract on different breast cancer cell lines. **A)** *P*. *lividus* gonadal extract effect on different breast cancer cell lines MDA-MB-231, MDA-MB-453, and HCC-1954. **B, C, D)** The extract inhibits the proliferation of MDA-MB-231, MDA-MB-453, and HCC-1954 cells, the effect was dose-dependent, 400 μg/ml and 600 μg/ml were the most effective doses. **E, F)** Gene expression of *Bcl2*, *ATM*, *ATR*, *BRCA1*, *CDK1*, *CDK4*, *CYCB1*, and *P21* in MDA-MB-231 and HCC-1954 breast cancer cell lines after treatment with *P*. *lividus* gonadal extract. Scale bar: 100 μm, invitro cell culture test, and RT-qPCR were performed in triplicates, significance: p ≤ 0.05.

To investigate the therapeutic potential of *P*. *lividus* gonadal extract, the present work assessed the viability of different breast cancer cell lines treated with an escalated extract dose. The triple-negative cell line MDA-MB-231, the androgen receptor-positive and triple-negative cell line MDA-MB-231, and the HER2-positive cell line HCC-1954 were utilized. The extract exerted concentration-dependent cytotoxic effects on the cell line tested; as shown in (Fig 2A–2D) Using non-linear regression analysis, The IC50 value of the extract in each cell line was estimated to be 360.8 μg/ml for MDA-MB-231, 379.8 μg/ml for MDA-MB-453 and 371.37 μg/ml for HCC 1954. Next, the effect of the gonadal extract on the expression patterns of a panel of genes involved in apoptosis and cell cycle regulation was investigated, as indicated in (Fig 2E and 2F), the investigated cells subjected to a sub-lethal extract dose exhibited a consistently significant downmodulation of the cyclin-dependent kinase 1 (CDK1) and its associated cyclin B1 (CYCB1). Concomitantly, the treated cells exhibited an upregulation of major cyclin-dependent kinase inhibitor p21. Taken together, the present data indicated a possible mode of action of the *P*. *lividus* gonadal extract by inhibiting cell proliferation and promoting cell cycle arrest.

### *P*. *lividus* gonadal extract possesses antimicrobial activity

**Fig 3:**
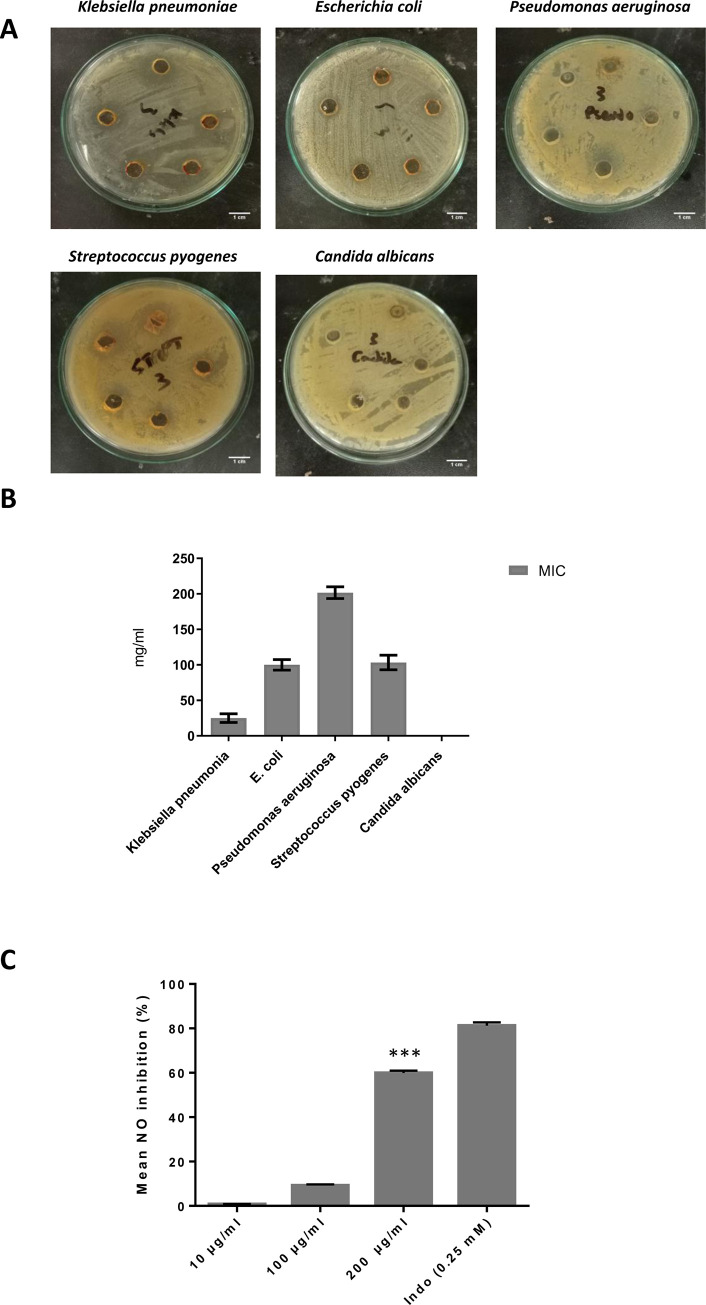
Antimicrobial and anti-inflammatory effect of *P*. *lividus* gonad extract on different microbial species and NO production in RAW 264.7 macrophage cell line. **A)** The inhibition zone of some Gram-positive, Gram-negative bacteria and *Candida albicans* to *P*. *lividus* gonadal extract. **B)** the minimum inhibition concentration of P. lividus gonadal extract for each microorganism. **C)** Anti-inflammatory activity of *P*. *lividus* gonadal extract different concentrations represented as NO% inhibition. Invitro antibacterial and anti-inflammatory tests were performed in triplicates, significance: p ≤ 0.05.

To examine the antimicrobial capacity of the extract, 5 pathogenic microbes with increasing concentrations of the extract were treated. Except for *Candida albicans* species, the extract demonstrated a potential antimicrobial effect assessed by measuring the inhibition zone diameter (Fig 3A & Table 4). Moreover, the minimum inhibitor concentrations (MIC) were calculated for the tested microorganisms where the gonadal extract possessed the lowest MIC against the Gram-negative bacteria *Klebsiella pneumoniae* and the highest MIC against *Pseudomonas aeruginosa* as shown in (Fig 3B).

**Table 4:** Antimicrobial effect of *P*. *lividus* gonadal extract against different species.

	Inhibition zone diameter (mm)**						
Strain	25 mg/ ml	50 mg/ ml	100 mg/ ml	150 mg/ ml	200 mg/ ml	Positive control	MIC
*Klebsiella pneumoniae*	11	15	17	18	20	38	25
*E*. *coli*	ND	ND	ND	ND	19	40	100
*Pseudomonas aeruginosa*	ND	ND	ND	ND	14	36	200
*Streptococcus pyogenes*	ND	ND	15	19	20	28	100
*Candida albicans*	ND	ND	ND	ND	ND	30	ND

**Diameters include 5 mm well diameter.

ND; Not detected.

MIC; Minimum inhibition concentration (mg/ml)

### *P*. *lividus* extract attenuates nitric oxide production in response to inflammation

The present study utilized the established murine macrophage RAW264.7 cell model to determine the anti-inflammatory effect of the gonadal extract. In this context, an inflammatory signal of 100 ng/ml of lipopolysaccharide (LPS) was induced either in the presence or absence of different concentrations of the extract. As a readout of the inflammatory response, the produced nitrate ions that represent the stable form of nitric oxide (NO) utilizing the Griess assay were measured. As demonstrated in (Fig 3C), at lower concentrations of the extract, no significant changes in the measured nitrate were observed. Nevertheless, at a higher concentration (200 μg/ml), the gonadal extract exhibited >50% inhibition of the stable NO form. Thus, the data indicated the potent effect of *P*. *lividus* gonadal extract in suppressing inflammatory signals, at least in murine macrophages.

## Discussion

In the present study, the GC-MS analysis of *P*. *lividus* gonadal extract showed a profile of different compounds with different bioactive properties. For instance, among the identified compounds, 1,2-Benzenedicarboxylic acid was reported earlier to inhibit the proliferation of hepatocellular carcinoma invitro cell line and arrest the cell cycle progression at the G1 phase [46]. Similarly, previous studies showed the ability of carotenoids to modulate the hallmarks of cancer mainly by inhibiting the cell cycle progression of actively dividing cancer cells [47]. Carotenoids were found to downmodulate cyclin D1, cyclin D2, CDK4, and CDK6 expression, and upregulate GADD45α, which prevents cell entry into the S phase [9]. Moreover, carotenoids induce the apoptotic potential of cancer cells and exhibit a synergetic effect when combined with conventional chemotherapeutic agents [48]. A previous study [49] attributed the broad inhibitory effect of carotenoids on cancer cells, including the anti-angiogenic and anti-metastasis effects, to the formation of retinoids from diverse carotenoids.

In a similar line, the gonadal extract of the *P*. *lividus* exhibited a significant reduction in cell proliferation of three different breast cancer cell lines. The gene expression analysis further reinforced the observed phenotype demonstrating a consistently significant downmodulation of crucial cell cycle regulators, namely CDK1 and CYCB1. On the other hand, gene expression analysis in the present study did not show any significant changes in CDK4 expression which regulates the entry to the G1 phase. Instead, the major regulators of the M-phase entry (CDK1/CYCB1) were significantly altered, pointing out a distinct mode of action. It is important to mention that the treated cells exhibited significantly elevated levels of the cyclin-dependent kinase inhibitor p21 compared to control cells. The p21 protein, also known as CDKN1A, was a direct target of p53 which was regulated at the transcriptional level upon genotoxic damage and stress conditions. Once stimulated, p21 induces a cell cycle arrest depending on the nature of the stimuli. Consistent with the present results, it is known that p21 inhibits the kinase activity of CDK1, causing a cell cycle arrest at the G2/M phase [50–52].

Carotenoids induce a cytotoxic effect on cancer cells by ROS induction without affecting the redox status and the proliferation of normal breast cells through the activation of pro-apoptotic proteins p21, p27, p53, and Bax, causing ROS-triggered apoptosis [53]. In this context, the Bcl2 family of proteins is known for its crucial role in regulating cellular apoptosis. A subfamily of Bcl-2, namely Bcl-2-like survival factors, acts as scavengers of pro-apoptotic proteins, performing anti-apoptotic functions [54, 55], also Bcl-2 caused mild prevention for the inflammation-induced cancer [56]. Blocking Bcl-2 mRNA translation reduced its protein level in the cells leading to apoptosis [57]. Previous studies demonstrated the pro-apoptotic functions of carotenoids by changing the expression levels of different Bcl-2 family proteins [58]. Thus, targeting Bcl-2 represents a potential therapeutic option to initiate apoptosis and overcome acquired resistance to several drugs [59, 60]. Interestingly, the investigated cell lines in the present study, MDA-MB-231, and HCC-1954 showed relatively lower expression levels of BCL-2 following the gonadal extract treatment. Adding to the activation of CDKN1A and downregulation of CDK1/CYCB1, the present data suggest a direct transcriptional dysregulation of key cell cycle and survival modulators that inhibit the proliferation capacity of cancer cells.

The comprehensive spectrum of anti-cancer properties inherent in 1,2-benzene dicarboxylic acid, mono(2-Ethylhexyl) ester was systematically investigated through an empirical exploration. The compound, isolated from Streptomyces sp., inhibited HepG2 cell proliferation in-vitro [46] and exhibited cytotoxic activity also on the MCF-7 cancer cell line while it showed less cytotoxic activity on murine fibroblasts and immortalized human keratinocytes NIH 3T3 and HaCa T, respectively [61]. In a parallel vein, extracts derived from gonadal tissues of the edible sea urchin *Heliocidaris erythrogramma* exhibited the capacity to suppress the proliferation of the mouse leukemia lymphoblastic cell line P388 [26].

To further examine the potential biological activities of *P*. *lividus* gonadal extract, an in vitro anti-inflammatory assay using RAW264.7 macrophage cell line was performed. Results revealed more than 50% inhibition of nitric oxide (NO) production in macrophages at 200 μg/ml extract concentration. In line with these results, previous reports indicated the potential anti-inflammatory effects of a carotenoid-enriched gonadal extract of different sea urchin species such as *Strongylocentrotus droebachiensis* and *Heliocidaris erythrogramma* (causing inhibition of the two isomers of bovine COX) [26, 28], *Stomopneustes variola*ris, *Echinometra mathaei* (causing inhibition of the protein denaturation) [12, 14].

In reference to the GC-MS and spectrophotometry results, the anti-inflammatory activity of *P*. *lividus* gonadal extract can, in part, be attributed to the presence of several bioactive compounds and carotenoids, respectively. For example, astaxanthin possesses prominent anti-inflammatory activity in different in vivo and in vitro model systems [62]. Additionally, in LPS-stimulated BV2 microglial cell inflammation, authors found that NO, iNOS and COX-2 expression were inhibited by astaxanthin [24, 63]. *β*-Carotene, Zeaxanthin, and Neoxanthin, detected in the carotenoid extract of guajillo peppers, were found to possess significant anti-inflammatory activity [64]. Moreover, numerous studies have reported the potent anti-inflammatory properties of PUFAs and palmitoleic acid (isomers of hexadecenoic acid) [65, 66].

*P*. *lividus* gonadal extract also showed anti-bacterial activity against all tested microorganisms. Similar findings were reported in other sea urchin species, such as *Diadema setosum* against *Klebsiella pneumoniae* [67], *E*. *mathaei* against streptococcus species [68], *Tripneustes gratilla* against *Escherichia coli* and *Pseudomonas aerogenes* [15]. The mechanistic proposal of the reported anti-bacterial activity of *P*. *lividus* gonad extract can be traced back to its content of palmitic acid, stearic acid, palmitoleic acid, and 9-cis-hexadecenoic as revealed by GC-MS analysis. In general, Fatty acids (saturated and unsaturated) have anti-bacterial activity against Gram-positive and Gram-negative bacteria [69, 70]. Also, hexadecenoic acid and octadecanoic acid were found to disrupt cytoplasmic membranes and induce DNA damage to *Staphylococcus aureus* and *Pseudomonas aeruginosa* [71]. Additionally, hexadecenoic acid, methyl ester inhibited the growth of *Escherichia coli*, *Pseudomonas aeruginosa* and *Klebsiella* bacterial strains [72, 73], whereas Pentadecane, Octadecane, and Eicosane possess anti-bacterial activity against the tested bacteria [74, 75]. Certain carotenoids induced lysozyme accumulation in the cell that digests bacterial cell walls [76] whereas, β-carotene and Astaxanthin inhibited *Pseudomonas aeruginosa* bacterial growth [77, 78] and Neoxanthin caused growth inhibition in helicobacter *pylori* growth [79]. According to previously published studies, lycopene inhibited bacterial growth by inducing reactive oxygen species (ROS)-mediated DNA damage in *Escherichia coli* [80] and restrained *Staphylococcus aureus*-induced inflammation by inhibiting the expression of α-hemolysin [81]. The biological activity of different compounds detected by GC-MS as reported by different literature is provided in (S1 Table) for the antibacterial, anti-inflammatory, antioxidant, anticancer and other biological properties [46, 66, 82–104].

In summary, the results of the current study provide preliminary experimental findings on the biological activity of *P*. *lividus* gonadal extract through a combination of in vitro anti-cancer, anti-inflammatory, and anti-bacterial assays. These findings support the integration of *P*. *lividus* gonadal extract into deeper biomedical investigations and to analytically isolate and identify promising marine natural lead compounds.

## Supporting information

S1 Fig *P*. *lividus* gonad extract compounds detected by GC-MS (A-R). Chemical structure, compound name, and reference library are provided for each compound.
(PPTX)

S1 Table Bioactivity of compounds detected by the GC-MS. (PPTX)
