# Supplementary material for: Characterization and biological applications of gonadal extract of Paracentrotus lividus collected along the Mediterranean coast of Alexandria, Egypt
Source: PLoS One. 2024 Jan 2;19(1):e0296312. doi: 10.1371/journal.pone.0296312 (PMC10760885; doi:10.1371/journal.pone.0296312)
Supplement: S1 Fig — P. lividus gonad extract compounds detected by GC-MS (A-R). Chemical structure, compound name, and reference library are provided for each compound. (PPTX) [file pone.0296312.s001.pptx]

## Slide 1
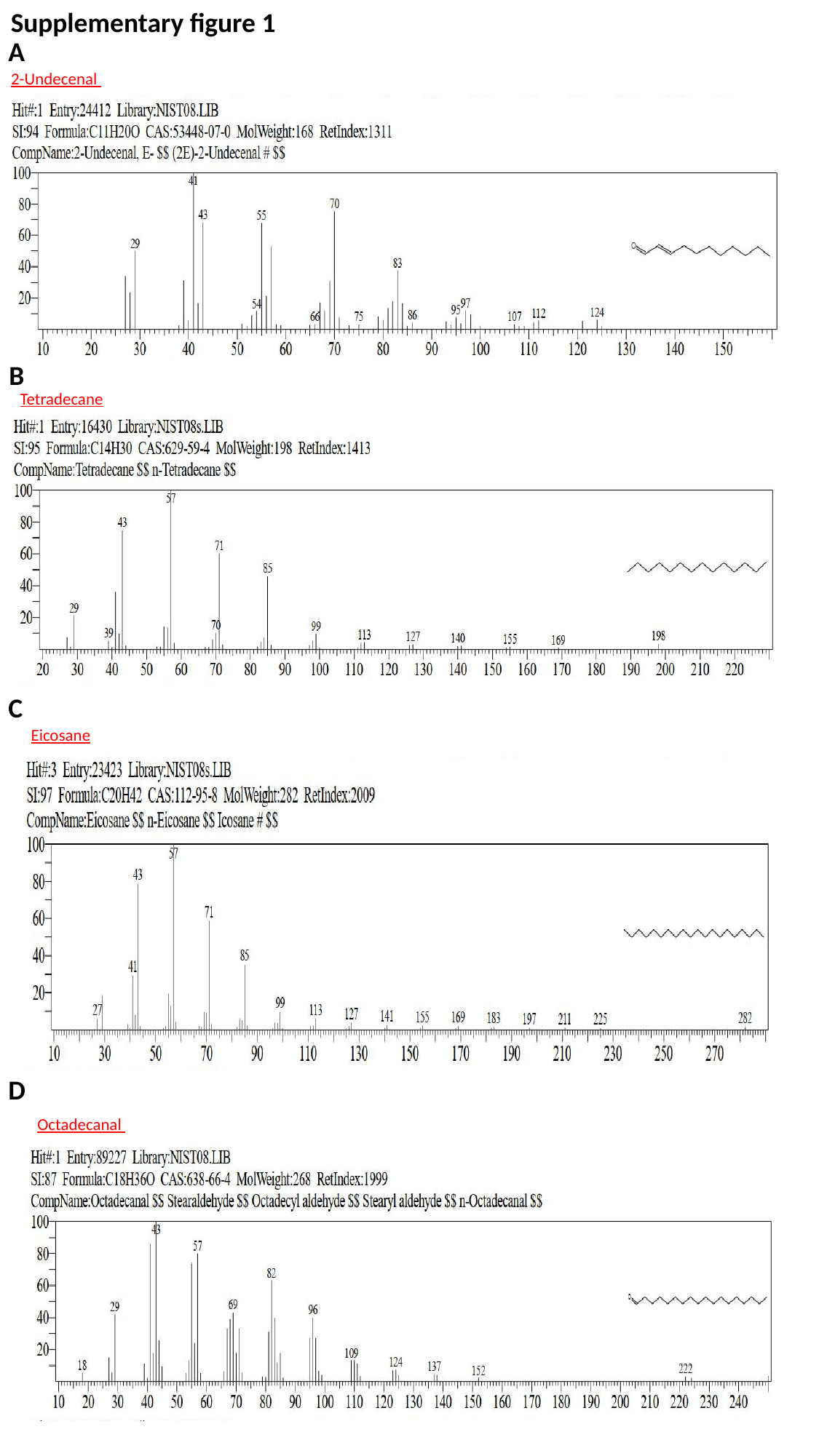

Supplementary figure 1
A
2-Undecenal
B
Tetradecane
C
Eicosane
D
Octadecanal

## Slide 2
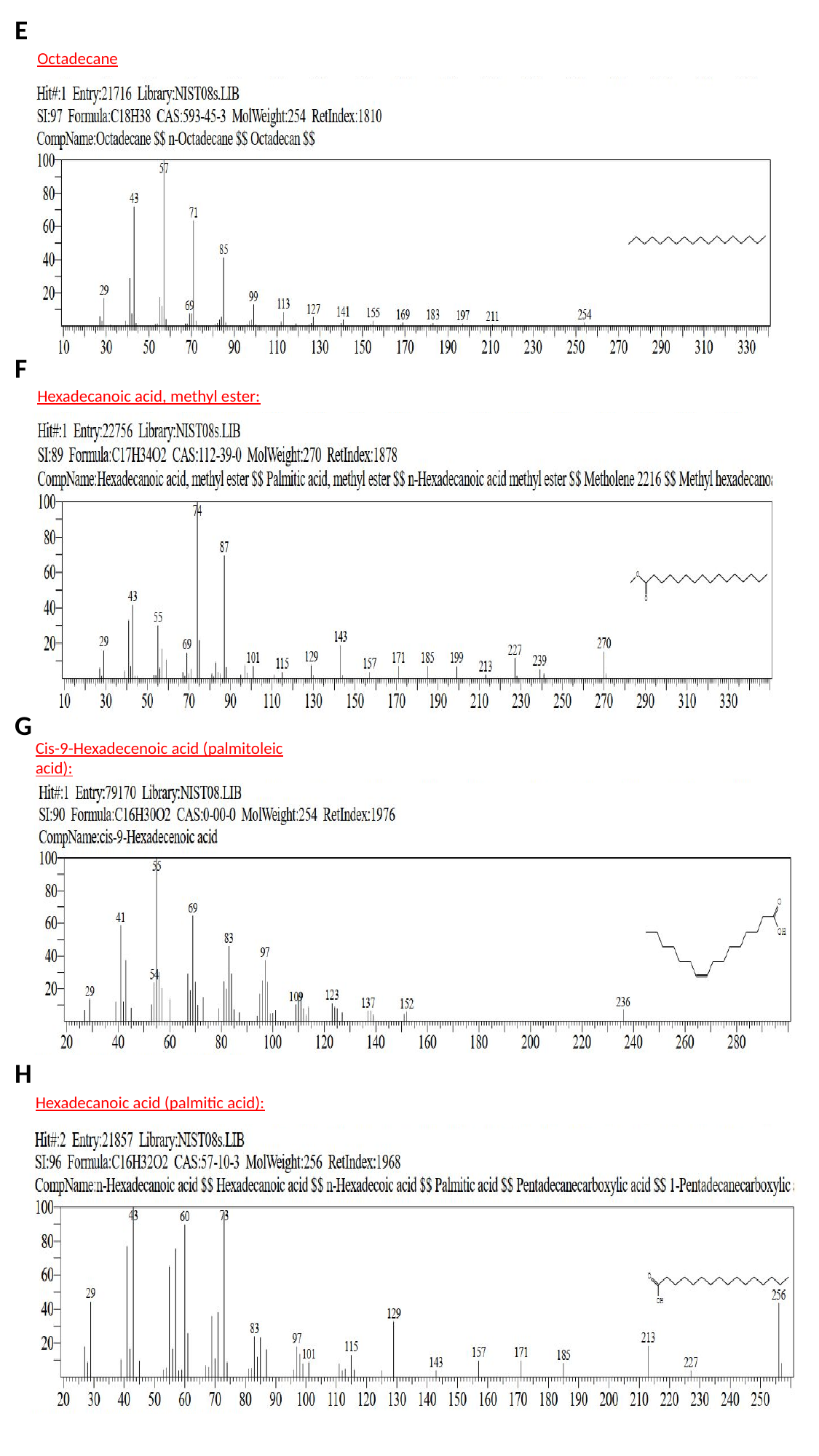

E
Octadecane
F
Hexadecanoic acid, methyl ester:
G
Cis-9-Hexadecenoic acid (palmitoleic acid):
H
Hexadecanoic acid (palmitic acid):

## Slide 3
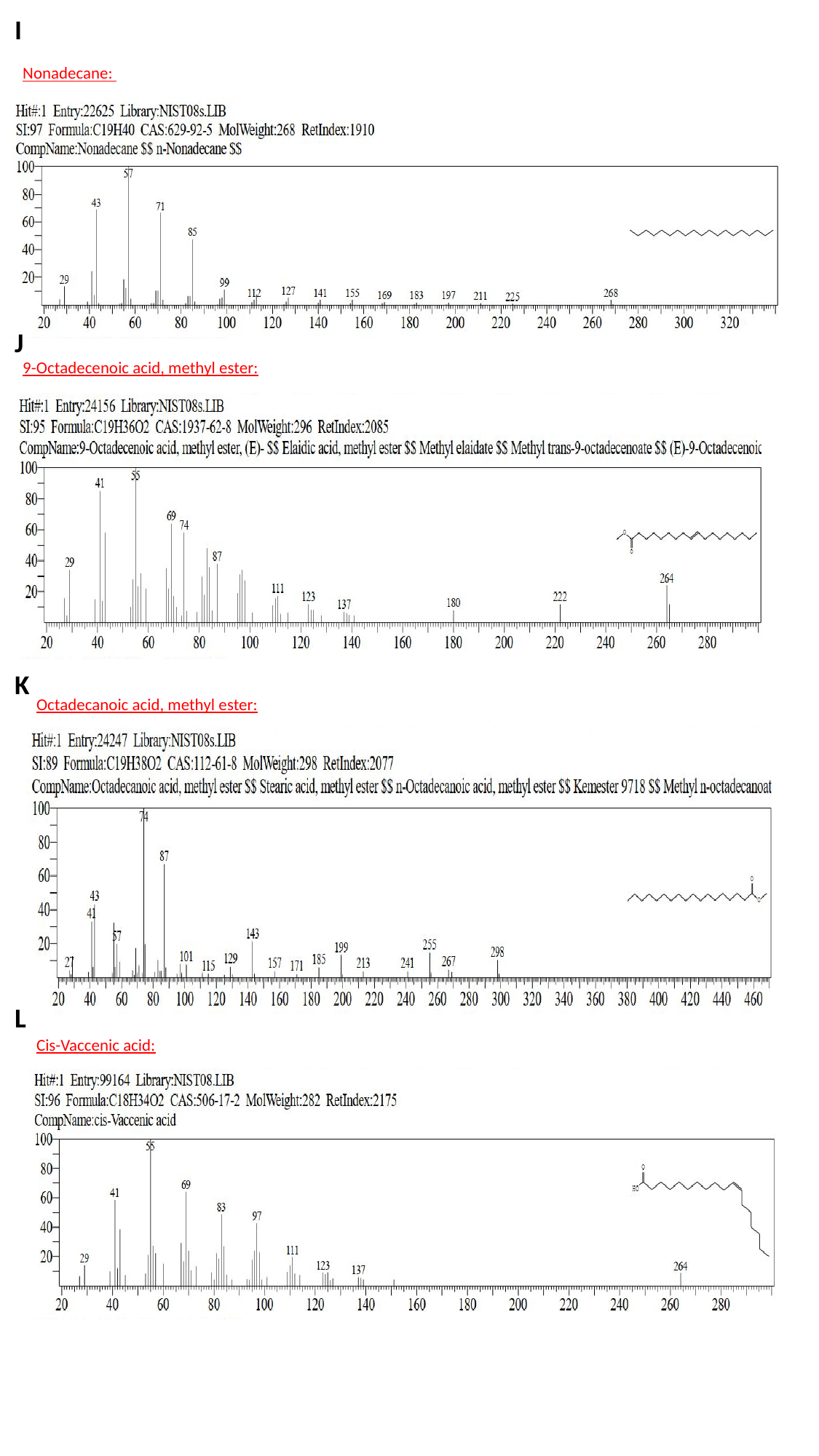

I
Nonadecane:
J
9-Octadecenoic acid, methyl ester:
K
Octadecanoic acid, methyl ester:
L
Cis-Vaccenic acid:

## Slide 4
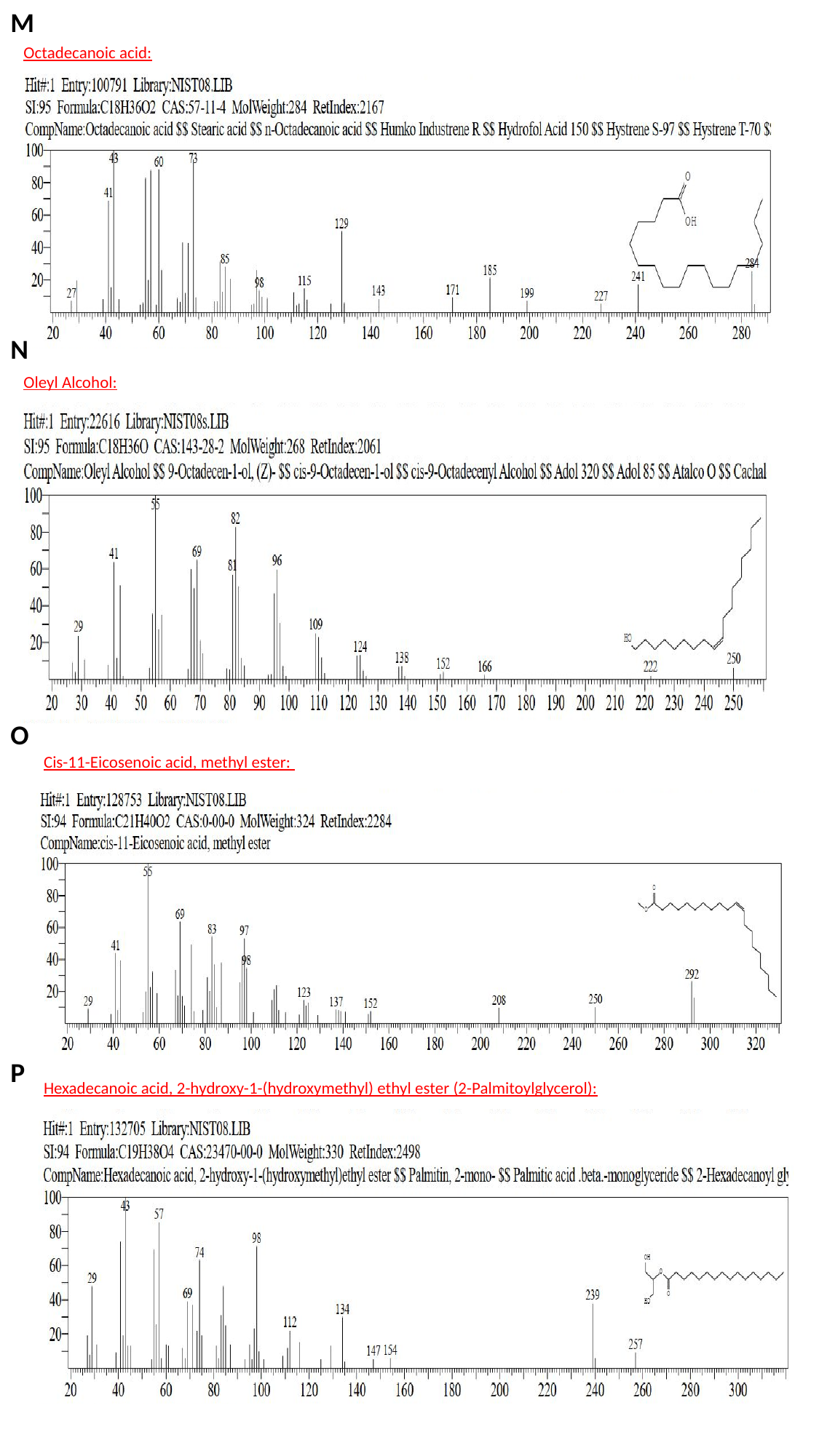

M
Octadecanoic acid:
N
Oleyl Alcohol:
O
Cis-11-Eicosenoic acid, methyl ester:
P
Hexadecanoic acid, 2-hydroxy-1-(hydroxymethyl) ethyl ester (2-Palmitoylglycerol):

## Slide 5
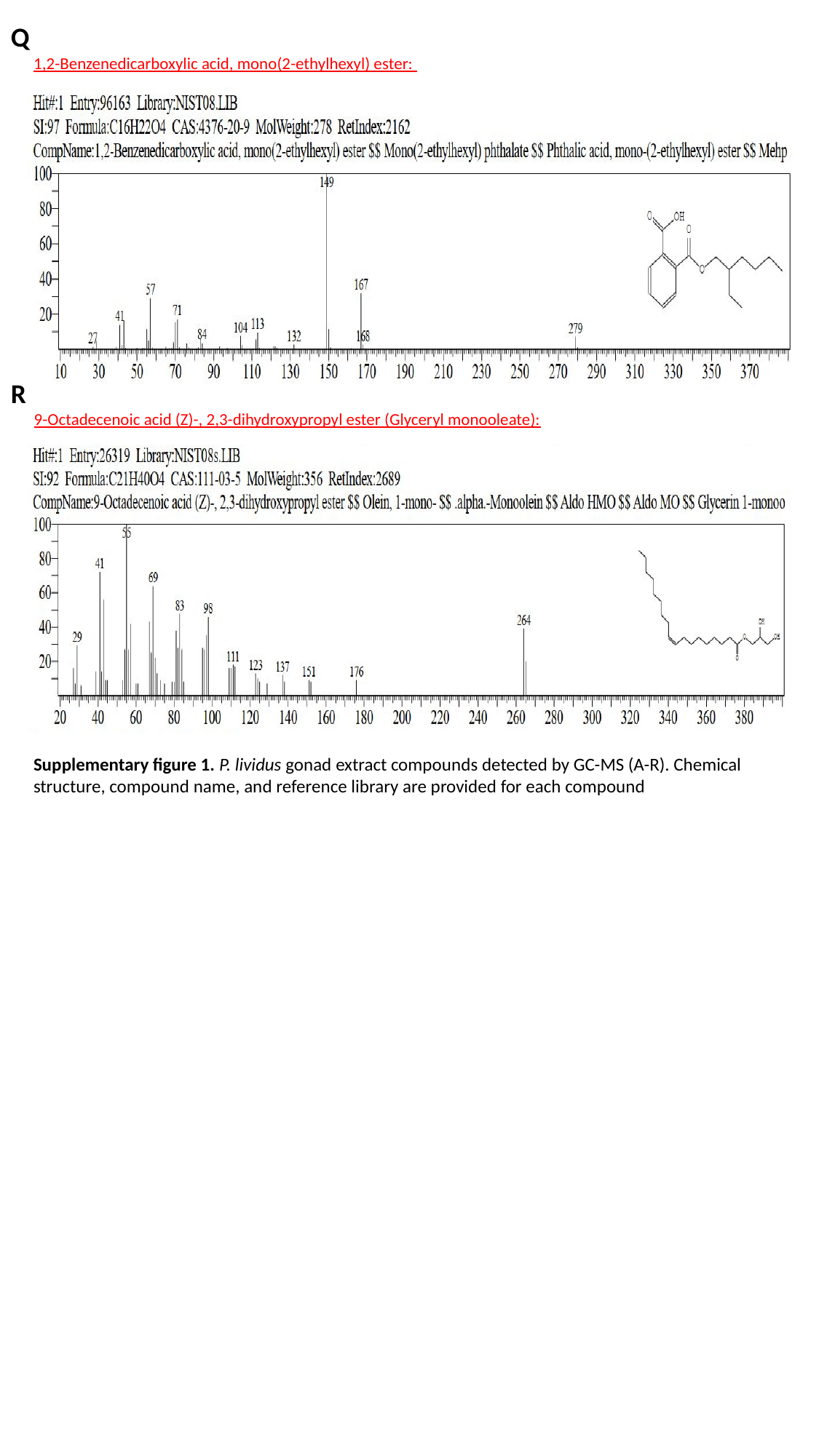

Q
1,2-Benzenedicarboxylic acid, mono(2-ethylhexyl) ester:
R
9-Octadecenoic acid (Z)-, 2,3-dihydroxypropyl ester (Glyceryl monooleate):
Supplementary figure 1. P. lividus gonad extract compounds detected by GC-MS (A-R). Chemical structure, compound name, and reference library are provided for each compound
