# Supplementary material for: Characterization and biological applications of gonadal extract of Paracentrotus lividus collected along the Mediterranean coast of Alexandria, Egypt
Source: PLoS One. 2024 Jan 2;19(1):e0296312. doi: 10.1371/journal.pone.0296312 (PMC10760885; doi:10.1371/journal.pone.0296312)
Supplement: S1 Table — (PPTX) [file pone.0296312.s002.pptx]

## Slide 1
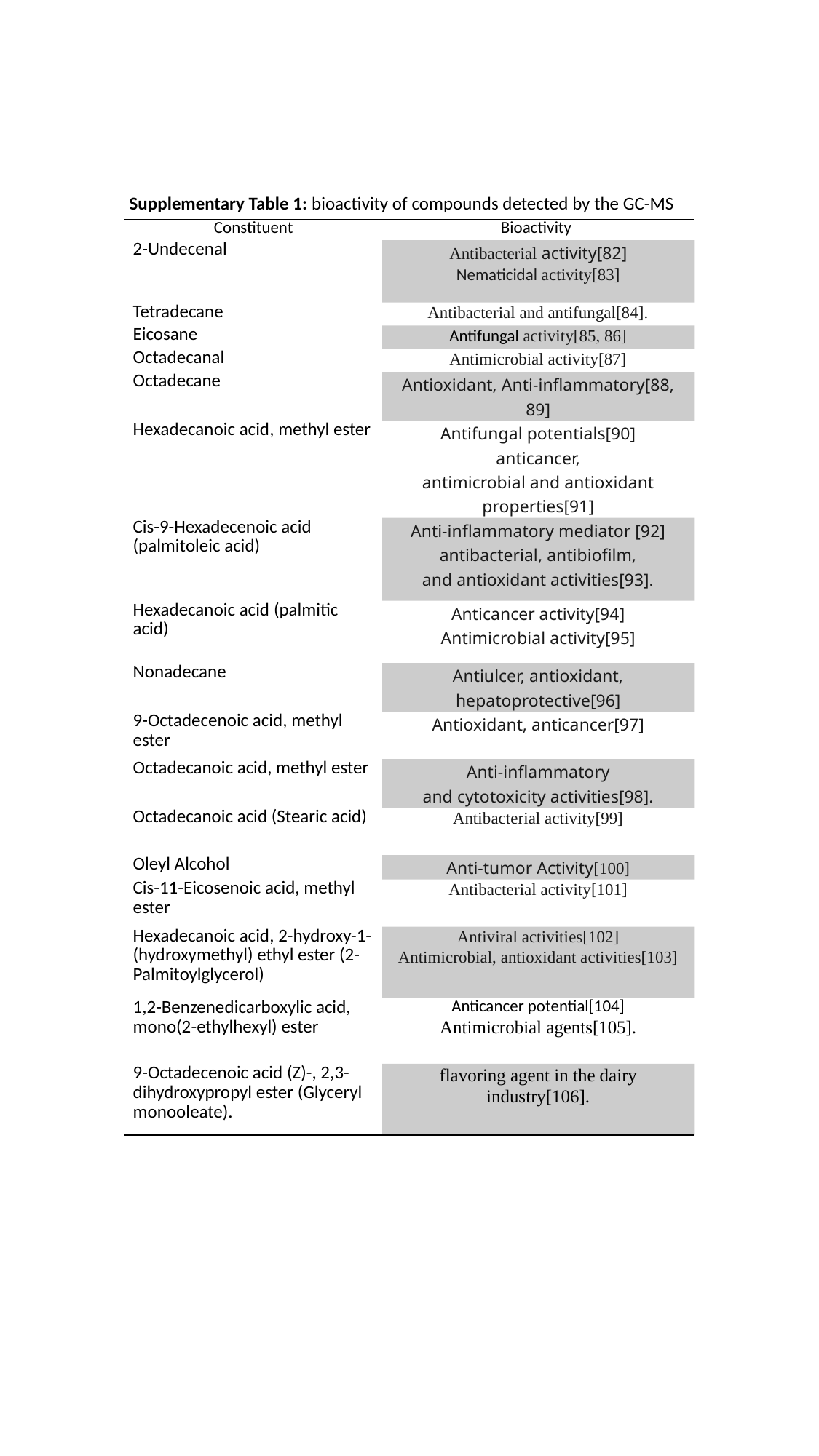

Supplementary Table 1: bioactivity of compounds detected by the GC-MS
| Constituent | Bioactivity |
| --- | --- |
| 2-Undecenal | Antibacterial activity[82] Nematicidal activity[83] |
| Tetradecane | Antibacterial and antifungal[84]. |
| Eicosane | Antifungal activity[85, 86] |
| Octadecanal | Antimicrobial activity[87] |
| Octadecane | Antioxidant, Anti-inflammatory[88, 89] |
| Hexadecanoic acid, methyl ester | Antifungal potentials[90] anticancer, antimicrobial and antioxidant properties[91] |
| Cis-9-Hexadecenoic acid (palmitoleic acid) | Anti-inflammatory mediator [92] antibacterial, antibiofilm, and antioxidant activities[93]. |
| Hexadecanoic acid (palmitic acid) | Anticancer activity[94] Antimicrobial activity[95] |
| Nonadecane | Antiulcer, antioxidant, hepatoprotective[96] |
| 9-Octadecenoic acid, methyl ester | Antioxidant, anticancer[97] |
| Octadecanoic acid, methyl ester | Anti-inflammatory and cytotoxicity activities[98]. |
| Octadecanoic acid (Stearic acid) | Antibacterial activity[99] |
| Oleyl Alcohol | Anti-tumor Activity[100] |
| Cis-11-Eicosenoic acid, methyl ester | Antibacterial activity[101] |
| Hexadecanoic acid, 2-hydroxy-1-(hydroxymethyl) ethyl ester (2-Palmitoylglycerol) | Antiviral activities[102] Antimicrobial, antioxidant activities[103] |
| 1,2-Benzenedicarboxylic acid, mono(2-ethylhexyl) ester | Anticancer potential[104] Antimicrobial agents[105]. |
| 9-Octadecenoic acid (Z)-, 2,3-dihydroxypropyl ester (Glyceryl monooleate). | flavoring agent in the dairy industry[106]. |
